# Introducing Group Open-Book Exams as a Learning and Assessment Strategy in the Clinical Biochemistry Course for Medical Students

**DOI:** 10.7759/cureus.51792

**Published:** 2024-01-07

**Authors:** Basmah Eldakhakhny, Aliaa A Alamoudi, Hoda Gad, Yousef Almoghrabi, Taghreed Shamrani, Hussam Daghistani, Abdulhadi Bima, Ghada Ajabnoor, Fayza Alfayez, Ayman Elsamanoudy

**Affiliations:** 1 Department of Clinical Biochemistry, King Abdulaziz University Faculty of Medicine, Jeddah, SAU; 2 Food, Nutrition, and Lifestyle Research Unit, King Fahd for Medical Research Centre, King Abdulaziz University, Jeddah, SAU; 3 Department of Medical Biochemistry and Molecular Biology, Alexandria University, Alexandria, EGY; 4 Department of Medical Biochemistry and Molecular Biology, Mansoura University Faculty of Medicine, Mansoura, EGY

**Keywords:** open-book exam, students' achievement, students' perception, clinical biochemistry, team-based learning

## Abstract

Background: Teachers constantly strive to obtain reliable and appropriate teaching and assessment methods to maximize the learning experience. This study aimed to introduce combined modified team-based learning and open-book exams (TBL/OBEs) as learning and assessment strategies in clinical biochemistry for medical students and assess students' perceptions.

Methods: Second-year medical students enrolled in the clinical biochemistry course were included in this study and subjected to TBL/OBE assessment. The assessment included two parts: the open-book format for half of the questions and the closed-book format for the other as a control. Upon completing the combined TBL/OBE session, the students were required to complete a structured survey to evaluate their perception of the experience. The data were gathered and analyzed. Data were presented as mean±standard error of the mean (SEM), and a p-value ≤0.05 was considered statistically significant.

Results: A total of 358 students completed the TBL/OBE and closed-book exam (CBE) and responded to the survey. Of these students, 76% preferred the OBE, and 84% thought it was a suitable learning method. On the one hand, the mean difficulty of the OBE format was 92.7±1.5 SEM, while, for the CBE, the mean difficulty was 88.7±1.9 SEM (p=0.015). On the other hand, the mean discrimination factor for OBE was 0.26±0.04 and, for the CBE, 0.41±0.04 SEM (p=0.0016). Males found the OBE questions easier (p=0.025) and less stressful (p=0.01).

Conclusion: A combined model of modified TBL and OBE is a successful learning and assessment strategy in clinical biochemistry for medical students.

## Introduction

Students everywhere find it difficult to study clinical biochemistry and challenging to retain the knowledge for an extended period and obtain an in-depth understanding that allows using the knowledge for problem-solving. Didactic lectures remain the primary teaching method in many medical educational facilities, including the Faculty of Medicine at the King Abdulaziz University (KAU) [1]. Concern regarding this issue has driven staff and other stakeholders to apply different teaching and learning modalities to enhance students' engagement, improve their performances, and increase their satisfaction. Examples of such teaching modalities are the introduction and evaluation of the team-based learning (TBL) strategy by Alamoudi et al. [1] and Alamoudi et al. [2] as well as the project-based learning (PrjBL) strategy by Elsamanoudy et al. [3]. In a quasi-experimental study at KAU, the learning outcomes and student satisfaction in the clinical biochemistry course were compared, with TBL being used for second-year clinical nutrition students and traditional lectures for same-year nursing students. The results indicated that students in the TBL group significantly outperformed those in the lecture group in final summative assessments. Moreover, 84% of students surveyed reported enjoying the TBL experience. The study further highlighted that accountability and teamwork were the principal positive elements recognized by students in the TBL approach [2].

Open-book exams (OBEs) have been introduced as a method of assessment that may enable students to achieve higher cognitive levels [4]. Compared to closed-book exams (CBEs), which rely on memorization, OBEs are considered a successful method to evaluate students' high-level skills based on Bloom's taxonomy, including conceptualizing, problem-solving, critical thinking, and reasoning [4]. Consequently, OBEs are regarded not only as an assessment method but also as a teaching and learning method that may foster deep learning and critical thinking and encourage self-directed learning [5]. However, the effect of OBEs compared to CBEs on students' performances is still debatable. In their meta-analysis conducted in 2013, Larwin et al. [6] found that open-book resources and testing improve exam performance, while Durning et al. [7] concluded that students perform and achieve better with CBEs than OBEs. Both discussed the effects on final-year medical students. Sam et al. [8] and Davies et al. [9] studied the effect of OBEs on academic performance in early-stage students (first and second years), and they revealed positive correlations between OBE and the students' academic achievement. This controversy shows that further research is warranted to explore best practices for designing and implementing OBEs to extract their full potential as an assessment and teaching method.

OBEs are based on several theoretical frameworks. One of the main theories underlying OBEs is constructivism [5], which emphasizes active learning and the construction of knowledge from various sources that can be applied to problem-solving. In addition, OBEs relate to cognitive load theory, where a reduction in demand on memory allows students to focus more on problem-solving and critical thinking [10,11].

TBL is an active learning strategy that has gained attention in medical education and has been implemented in several courses across medical curricula [12]. The preparation phase of TBL ensures that students (individually) obtain new information through reading and understanding the provided material. Thus, students are accountable for their learning process. One of the most critical elements of TBL is teamwork. During the readiness phase, a test on the material is first completed by each student individually and then in groups that have to reach a consensus on the answers. To reach agreement while answering the test, students have to learn to listen to each other, communicate and negotiate, discuss concepts, and jointly construct their understanding, all of which require effective group interaction. Working in groups also enables students to engage more with their material [13]. In addition, knowledge gaps are exposed during team discussions, and peers explaining to their team members aid in addressing these gaps [14]. One of the main aspects of TBL that supports collaborative learning is that teams are not constructed randomly but rather strategically to ensure a diversity of skills and levels to maximize the learning experience. In contrast, students tend to work solo during outcome-based education (OBE) sessions, which diminishes the potential for collaborative learning and social interdependence. There is substantial evidence indicating that collaborative learning, especially in small groups where members collectively strive toward shared educational objectives, enhances knowledge retention, critical thinking, and interpersonal skills. Teaching strategies like problem-based learning (PBL) and TBL have heavily emphasized collaborative learning [15,16]. Therefore, integrating elements of TBL, such as teamwork and collaboration, into OBE could significantly boost student performance and engagement.

In this study, we introduce a collaboratively modified TBL/OBE as a learning and assessment method for second-year medical students enrolled in clinical biochemistry. The study aims to examine the correlation between student performance in the modified TBL/OBE and their overall clinical biochemistry academic performance, to investigate students' perception of the effectiveness of this new teaching and assessment strategy by filling out a questionnaire assessing the learning materials and the implementation of TBL and OBE, and to evaluate the effectiveness of teamwork. Through exploring these aspects, the study seeks to provide insights into the effectiveness of modified TBL/OBE as a pedagogical tool and its implications for enhancing student learning experiences, specifically in cultures where active teaching and learning strategies are not widely applied.

## Materials and methods

Participants

All 418 KAU second-year medical students in the 2021-2022 academic year were included in this study. Students study clinical biochemistry (BCHM 201) as a core course during the first semester of phase 1 basic sciences courses at the Faculty of Medicine, King Abdulaziz University, Saudi Arabia. It is a five-credit course with seven contact hours per week. This study was approved by the Biomedical Ethics Research Committee of King Abdulaziz University (approval number: 93-22). The questionnaire was available for one day at the end of the TBL session, and all students consented to participate before completing the questionnaire.

Implementation and preparation

The study was conducted at the conclusion of the clinical biochemistry course while teaching the Integration and Metabolic Interrelation chapter. Before the implementation phase, students were assigned pre-class reading and offered lecture notes to support our study's aim of examining TBL/OBE as a learning strategy, ensuring they had essential references and learning material. The lectures started with the expected students' learning outcomes, followed by a detailed description of the topic's content. In addition, a video related to the subject prepared by the instructor was also presented. The students were encouraged to study the required references (Lippincott Illustrated Reviews, 6th edition, Champe & Harvey, unit chapters 23 and 24, and Medical Biochemistry, Baynes and Dominiczek, 1st edition, Mosby).

Furthermore, we encouraged the students to revisit all previous lectures on metabolism (Met-Lec), and some students preferred to revise using YouTube (YT) channels. Finally, a formative assessment containing a sample of assessment questions with model answers was provided to the students. All the materials were uploaded to the online blackboard system 10 days prior to the in-class TBL/OBE session to accommodate students' schedules, allowing them to balance other academic responsibilities and to go through the learning cycle of comprehension, application, and analysis.

Students were divided into 69 teams (34 male teams and 35 female teams) consisting of five/six students each. Each group comprised students representing every grade level, A, B, C, D, and F, to ensure a uniform skill level across the groups. Prior to the OBE, students participated in a midterm exam and three quizzes. Their cumulative scores from these assessments were utilized to assign students into groups randomly.

Implementation of combined TBL and OBE

Orientation Session

Since the students were new to the TBL/OBE process, a blackboard orientation session was planned and delivered. The TBL/OBE process was explained using PowerPoint, and an open discussion was allowed. The students had already been informed about the formation of the teams 10 days previously and were assigned to their respective teams. They were instructed to select a team leader and an appropriate name for their group. This session was intended as an activity for the teams.

Combined TBL/OBE Session

The TBL/OBE session was conducted on campus. Each group was in small classrooms with a round table. Two separate assignments had been prepared by the instructors in advance. Each assignment consisted of eight multiple-choice questions, one essay question, and one extended matching question. These assignments included questions that required hypothetical thinking, comprehensive matching, and reasoning and challenged the students to apply, analyze, synthesize, and/or evaluate. After each multiple-choice question, a blank section was left so the students could explain their choices. The essay responses were evaluated using a structured answer key rubric containing specific keywords aimed at assessing content comprehension, critical thinking, analytical skills, and organization. The session consisted of two sections. During the first one, the students had to discuss and answer the questions without any resources, described hereafter as a CBE. In contrast, during the second section, the students were asked to discuss and answer the questions with an open book and open web. Each team had to provide their answers in a separate booklet for each session (one for the CBE and another for the OBE). The students were encouraged to promote discussion and encourage teamwork to reach a consensus. The groups were divided into Groups A and B. Group A received Q1-10 in the CBE format and then Q11-20 in the OBE format, and vice versa for Group B. The students had two hours to complete the test, one hour for each set of questions, followed by a feedback session where all questions were answered and interactively discussed by the instructor and the students.

We considered this approach a modified TBL, focusing only on in-class application activity. Each team worked on the same significant problem but lacked readiness assurance testing (individual Readiness Assurance Testing (iRAT) and team Readiness Assurance Testing (tRAT)).

The survey

After completing the combined TBL/OBE sessions, the students were required to complete a structured survey (Google Forms) comprising 26 items. The authors prepared the survey, revised it, and validated it with the aid of local and national experts in medical education.

The form contained three sections. The first section assessed the usefulness of the reading materials, videos related to the topic, text, and facilitator discussion. The second section elicited their feedback on the implementation of TBL and OBE. The third section was intended to assess the effectiveness of teamwork. It included items that evaluate the students' passion, commitment to the team, interpersonal relationships, and communication. The students recorded their responses on a modified five-point Likert scale.

Statistical analysis

Data analysis was done using IBM SPSS Statistics for Windows, Version 26.0 (Released 2019; IBM Corp., Armonk, New York, United States), and GraphPad Prism, Version 9.5 (GraphPad Software, Boston, Massachusetts, United States, www.graphpad.com). Chi-squared analysis was done to compare males and females in different grade groups. An analysis of variance (ANOVA) test was done to compare different grade groups, followed by a post-hoc Tukey test. The p-value was calculated with the use of a paired t-test to compare the difficulty and discrimination of the questions. Data are presented as mean±standard error of the mean (SEM), and the p-value is considered significant if <0.05.

## Results

Students' preparation

**Figure 1:**
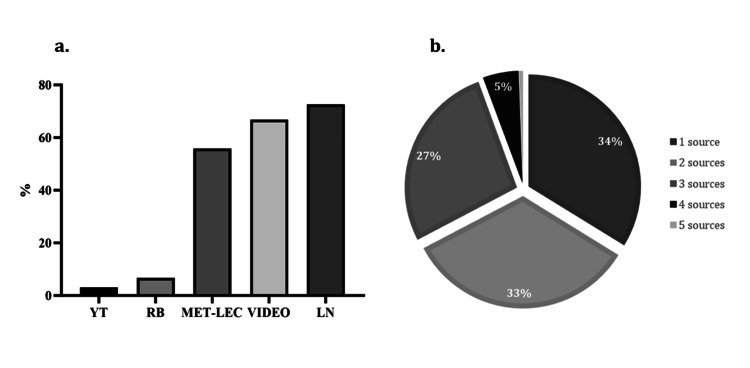
Exam preparation resources. a) Different resources were offered to students to prepare for TBL/OBE. 72% used the integration of metabolism lecture notes and video recordings, and 67% studied all metabolism lectures. b) Students combined different resources in preparation, with the majority combining two or more of the following methods: YT, RB, Met-Lec, videos (integration and interrelation of video recordings), and LN. TBL/OBE: team-based learning and open-book exam; YT: YouTube; RB: reference book; Met-Lec: all metabolism lectures; LN: integration and interrelation of lecture notes

Of the 418 students who participated in the OBE/CBE sessions, a total of 358 (185 females (52%) and 173 males (48%)) completed the survey. Students had 10 days to prepare for the exam. Of the students, 94% familiarized themselves with the OBE rules and regulations provided by the department, and 54% had previous experience with the OBE format. Most students revised with lecture notes and video recordings, 72% and 67%, respectively, and 55% studied all metabolism-related lectures (Figure 1).

Furthermore, 34% of the students used only one resource type, 33% used two, and 27% used three (Figure 1b). Table 1 summarizes the students' perceptions of the time and materials provided to prepare for the exam. Most students found that the provided materials and videos were beneficial, ranging from a 3 to 5 Likert scale. On the other hand, the majority felt that the time to prepare for the exam was insufficient.

**Table 1:** Students' perception of OBE preparation. The numbers represent median, minimum, and maximum score of the item per survey question. OBE: open-book exam

Questions	Median	Minimum	Maximum
Did you benefit from the lecture video recording? (1 strongly disagree, 5 strongly agree)	5	1	5
Did you find the objectives and materials given to you clear? (1 not clear, 5 very clear)	3	1	5
Did you find preparation for OBE less stressful than a CBE? (1 less stressful, 5 very stressful)	3	1	5
Did you need more time to prepare for the OBE? (1 strongly disagree, 5 strongly agree)	5	1	5

Students' perception of OBE and teamwork

**Figure 2:**
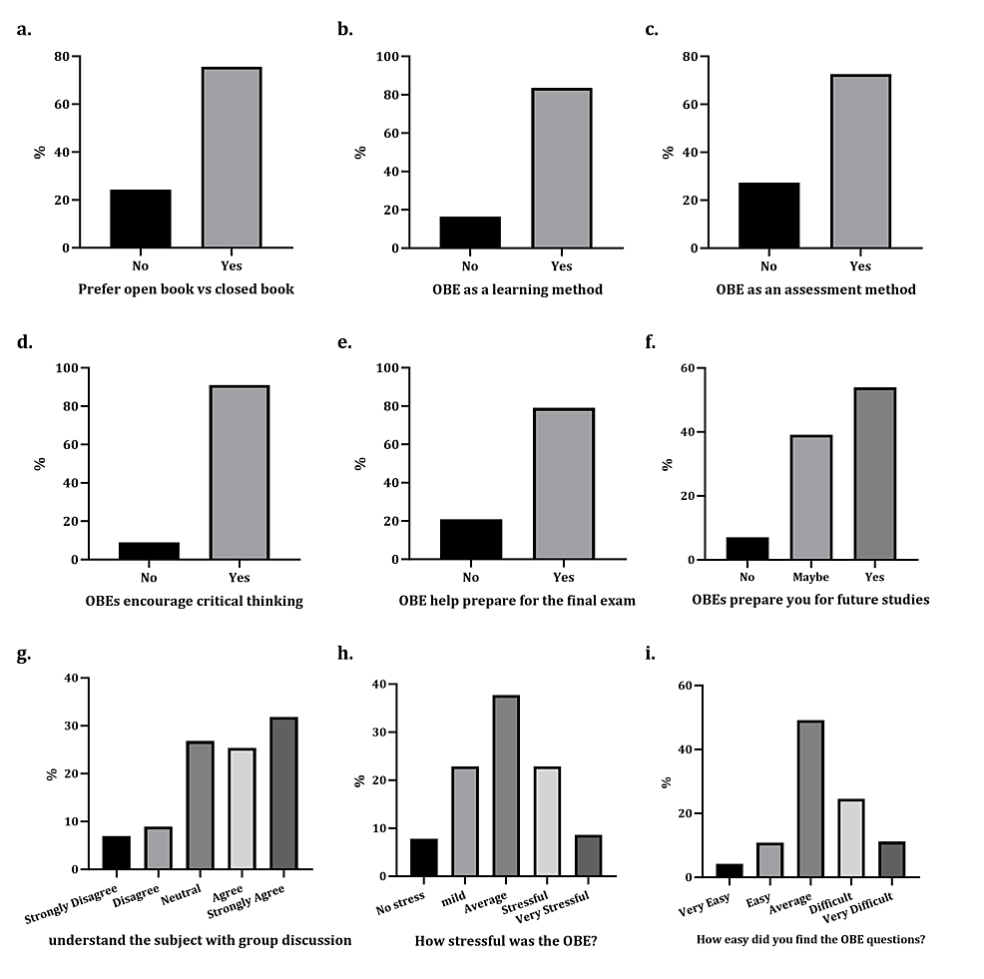
Students' perception of OBE. The students' perception of the OBE is illustrated as follows: a) students prefer OBEs versus CBEs, b) the use of OBE as a learning and c) assessment method, d) how OBEs encourage critical thinking, e) how OBE helps prepare for the final exam, f) the extent to which OBEs prepare students for future studies, g) the extent to which OBEs help students to understand the subjects, h) how stressful, and i) how easy. OBE: open-book exam; CBE: closed-book exam

After the open- and closed-format exams, the students completed a verified questionnaire related to their perceptions of OBE versus CBE and their experience of teamwork. Of the students, 76% preferred the open-book format (Figure 2a), 84% regarded it as a suitable learning method (Figure 2b), and 73% would prefer to have more assessments in the form of OBEs (Figure 2c). Furthermore, 91% of students stated that OBEs encourage critical thinking (Figure 2d), 79% found it helpful to study for the final exam (Figure 2e), 54% thought that this format would be useful in future studies (Figure 2f), and 55% of the students found team discussions beneficial for a better understanding of the subject (Figure 2g). Moreover, 37% experienced an average stress level during the OBE; however, around 30% perceived it as stressful (Figure 2h), and 50% found the difficulty of the questions to be average (Figure 2i).

**Figure 3:**
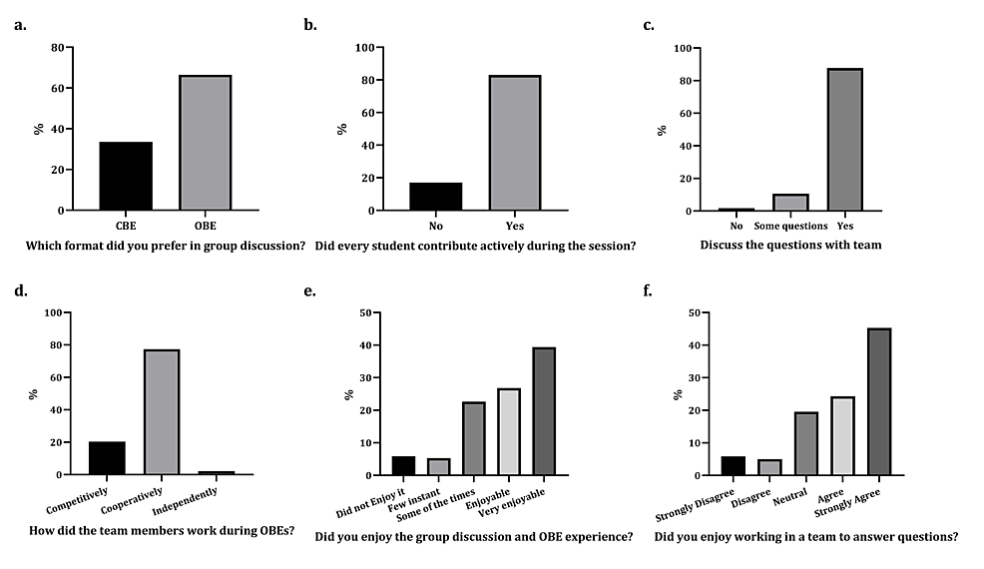
Students' perception of teamwork and modified TBL. The students' perception of the implemented modified TBL and the teamwork is illustrated as follows: a) Which format did you prefer in group discussion? b) Did every student contribute actively during the session? c) Did you discuss the questions' answers with your team? d) How did the team members work during OBEs (competitive, cooperative, or independent)? e) Did you enjoy the group discussion and OBE experience? f) Did you enjoy working in a team to answer questions? TBL: team-based learning; OBE: open-book exam

The students preferred working in a group in the OBE format to working in a group in the CBE format, 67% and 33%, respectively (Figure 3a). Of the students, 83% contributed equally to the group activity and actively discussed the questions within the group (Figure 3b, Figure 3c), 77% answered the questions cooperatively (all team members collaborated to answer all the questions), 20% worked competitively (competing with team members as to who could produce the best answer for a specific question), and 3% preferred to divide the questions among the group members and answer alone (Figure 3d). Finally, most students enjoyed group discussions, working in a team, and the overall OBE experience (Figure 3e, Figure 3f).

OBE vs CBE scores

**Figure 4:**
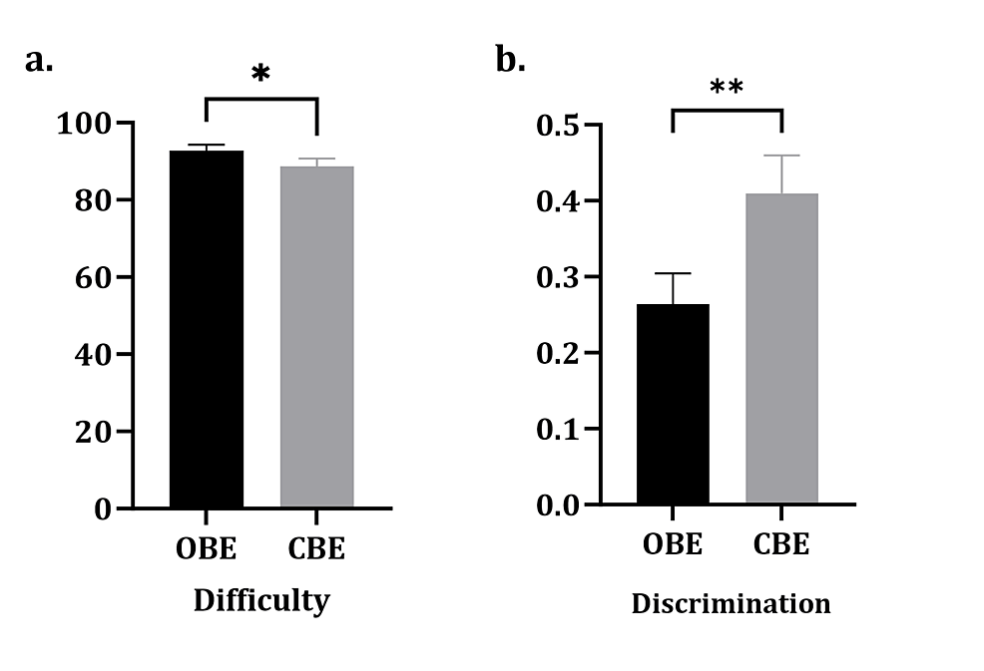
The difference between the difficulty index and the discrimination factor is administered as an open book or blind format. The average difficulty and discrimination were calculated for all questions. a. The difficulty index. b. The difference in discrimination factor with the questions in a CBE format had the higher discrimination. Data were presented as mean±SEM; paired t-test was calculated. *p-value <0.05. **p-value <0.01. CBE: closed-book exam; SEM: standard error of the mean

Students were placed in a group of 5 (each group had one student from a different grade level, A-F, based on their clinical biochemistry grade) and then distributed into Groups A and B. Question difficulty index and discrimination factors were calculated for both formats (Figure 4). On the one hand, the OBE mean difficulty was 92.7±1.5 SEM, while the CBE mean difficulty was 88.7±1.9 SEM (p=0.015). On the other hand, the mean discrimination factor for the open-book format was 0.26±0.04 and 0.41±0.04 SEM for the CBE format (p=0.0016).

The perception of students in different grade levels of OBE

The students' answers, organized according to their final grade (A-F) in the biochemistry course, are presented in Table 2 and Table 3. The grades underwent analysis using an ANOVA test (Table 2), followed by a post-hoc test for any significant findings, to identify the groups with notable differences precisely (Table 3). There was a statistical difference between how they perceived the difficulty of the questions (p-value between the groups: 0.006). The differences were between the students with A, C, and D grades. Interestingly, compared to students with lower grades, a high percentage of students with B grades found the exam extremely difficult. The majority of the students preferred the OBE format, but the highest number of students who chose the CBE format were students with A and B grades.

**Table 2:** Students in different grade levels have different perceptions of OBE. Bold values indicate a p-value of <0.05. OBE: open-book exam; ANOVA: analysis of variance

Questions		A (n, %)	B (n, %)	C (n, %)	D (n, %)	F (n, %)	X^2^ p-value	ANOVA p-value
How easy did you find the OBE questions?	Very easy	3 (4.8%)	8 (6.1%)	3 (3.3%)	0 (0%)	1 (3.3%)	0.019	0.0061
	Easy	8 (12.9%)	15 (11.5%)	13 (14.1%)	2 (4.7%)	1 (3.3%)		
	Average	41 (66.1%)	53 (40.5%)	45 (48.9%)	20 (46.5%)	17 (56.7%)		
	Difficult	6 (9.7%)	39 (29.8%)	25 (27.2%)	11 (25.6%)	7 (23.3%)		
	Very difficult	4 (6.5%)	16 (12.2%)	6 (6.5%)	10 (23.3%)	4 (13.3%)		
Would you prefer to have more OBE as an assessment method?	No	27 (43.5%)	34 (26%)	21 (22.8%)	12 (27.9%)	4 (13.3%)	0.016	0.0152
	Yes	35 (56.5%)	97 (74%)	71 (77.2%)	31 (72.1%)	26 (86.7%)		
How did the team members work during OBEs?	Competitively	9 (14.5%)	32 (24.4%)	16 (17.4%)	11 (25.6%)	5 (16.7%)	0.394	0.2886
	Cooperatively	51 (82.3%)	96 (73.3%)	75 (81.5%)	32 (74.4%)	23 (76.7%)		
	Independently	2 (3.2%)	3 (2.3%)	1 (1.1%)	0 (0%)	2 (6.7%)		
Which format did you prefer in group discussions?	Closed	27 (43.5%)	51 (38.9%)	21 (22.8%)	15 (34.9%)	6 (20%)	0.019	0.0189
	Open	35 (56.5%)	80 (61.1%)	71 (77.2%)	28 (65.1%)	24 (80%)		
Did the group members understand metabolic integration and interrelation clearly?	No	14 (22.6%)	31 (23.7%)	25 (27.2%)	5 (11.6%)	5 (16.7%)	0.311	0.3138
	Yes	48 (77.4%)	100 (76.3%)	67 (72.8%)	38 (88.4%)	25 (83.3%)		
Did you enjoy the group discussion and OBE experience?	Did not enjoy it	5 (8.1%)	7 (5.3%)	5 (5.4%)	3 (7%)	1 (3.3%)	0.922	0.7268
	Few instants	2 (3.2%)	10 (7.6%)	5 (5.4%)	2 (4.7%)	0 (0%)		
	Some of the times	19 (30.6%)	25 (19.1%)	21 (22.8%)	8 (18.6%)	8 (26.7%)		
	Enjoyable	16 (25.8%)	34 (26%)	25 (27.2%)	12 (27.9%)	9 (30%)		
	Very enjoyable	20 (32.3%)	55 (42%)	36 (39.1%)	18 (41.9%)	12 (40%)		
Did you enjoy working in a team to answer questions?	Strongly disagree	5 (8.1%)	13 (9.9%)	0 (0%)	2 (4.7%)	1 (3.3%)	0.418	0.1630
	Disagree	3 (4.8%)	7 (5.3%)	5 (5.4%)	2 (4.7%)	1 (3.3%)		
	Neutral	15 (24.2%)	23 (17.6%)	18 (19.6%)	10 (23.3%)	4 (13.3%)		
	Agree	17 (27.4%)	27 (20.6%)	27 (29.3%)	9 (20.9%)	7 (23.3%)		
	Strongly agree	22 (35.5%)	61 (46.6%)	42 (45.7%)	20 (46.5%)	17 (56.7%)		

**Table 3:** Post-hoc analysis for the student in different grade levels. OBE: open-book exam

Questions	Grade	Grade	p-value
How easy did you find the OBE questions?	D	F	0.73
		C	0.045
		B	0.163
		A	0.003
Would you prefer an open-book assessment as compared to a closed-book assessment method?	A	F	0.091
		D	0.076
		C	0.01
		B	0.143
Would you prefer to have more OBE as an assessment method?	A	F	0.019
		D	0.383
		C	0.036
		B	0.075

Furthermore, even though the majority of the students preferred OBE as an assessment method, the students with A grades had the lowest percentage, 54%, compared to 72-86% among the students with lower grades. A high percentage of students who preferred to work competitively had B grades, but there was no statistical significance. Finally, fewer students with A grades enjoyed the OBE experience and teamwork compared to the other grade groups.

Differences between males and females in their OBE perception

On examining the differences in the experience of males and females (Table 4), we found that the males found the OBE questions easier (p=0.025) and less stressful (p=0.01) and that they needed less time to prepare for the exam (p=0.014). A higher percentage preferred OBEs as learning and assessment methods. During the OBE, the majority of the students, both males and females, worked cooperatively, but interestingly, 24% of the females worked competitively versus 16% of the males, and 4% of the males preferred to work independently versus 0.5% of the females (p=0.018).

**Table 4:** Difference between males and females. OBE: open-book exam

Question		Male	Female	X^2^ p-value
		(n, %)	(n, %)	
How easy did you find the OBE questions?	Very easy	8 (4.6%)	7 (3.8%)	0.025
	Easy	27 (15.6%)	12 (6.5%)	
	Average	84 (48.6%)	92 (49.7%)	
	Difficult	41 (23.7%)	47 (25.4%)	
	Very difficult	13 (7.5%)	27 (14.6%)	
How stressful was the OBE?	No stress	13 (7.5%)	15 (8.1%)	0.01
	Mild	52 (30.1%)	30 (16.2%)	
	Average	59 (34.1%)	76 (41.1%)	
	Stressful	40 (23.1%)	42 (22.7%)	
	Very stressful	9 (5.2%)	22 (11.9%)	
Would you prefer to have more OBE as a learning method?	No	21 (12.1%)	38 (20.5%)	0.032
	Yes	152 (87.9%)	147 (79.5%)	
Would you prefer to have more OBE as an assessment method?	No	38 (22%)	60 (32.4%)	0.026
	Yes	135 (78%)	125 (67.6%)	
Did OBEs help you prepare for the final exam?	No	25 (14.5%)	50 (27%)	0.003
	Yes	148 (85.5%)	135 (73%)	
How did the team members work during OBEs?	Competitively	28 (16.2%)	45 (24.3%)	0.018
	Cooperatively	138 (79.8%)	139 (75.1%)	
	Independently	7 (4%)	1 (0.5%)	
Did you need more time to prepare for the OBE?	Strongly disagree	9 (5.2%)	5 (2.7%)	0.014
	Disagree	20 (11.6%)	18 (9.7%)	
	Neutral	52 (30.1%)	51 (27.6%)	
	Agree	50 (28.9%)	36 (19.5%)	
	Strongly agree	42 (24.3%)	75 (40.5%)	

Correlation of the students' performance in TBL/OBE and the overall performance in the clinical biochemistry course

**Figure 5:**
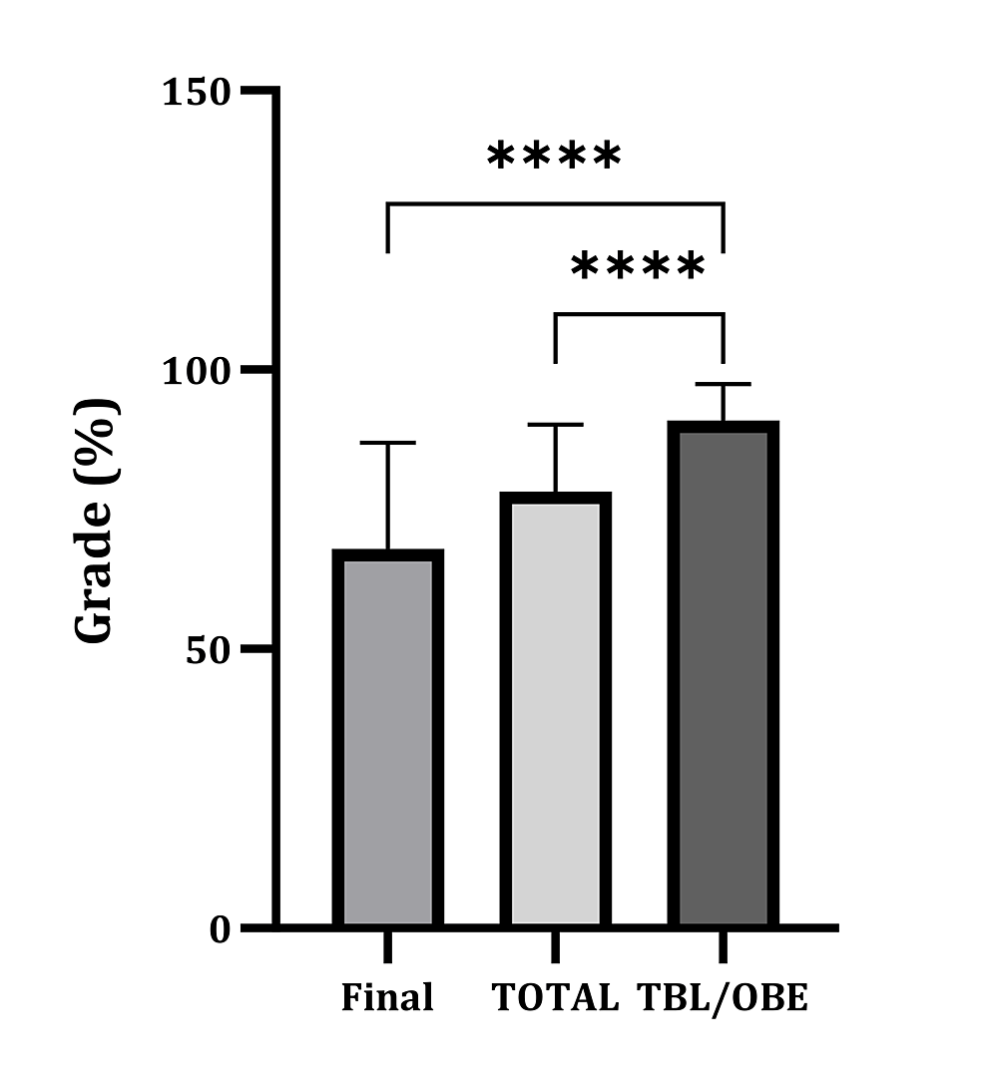
The difference between the students' academic achievement in TBL/OBE, final, and total results. Data are presented as mean±SD. Paired t-test was calculated. ****p-value <0.0001. TBL/OBE: team-based learning and open-book exam; SD: standard deviation

The students' performance in TBL/OBE positively correlates with their academic achievement in the final exam and overall score in the clinical biochemistry results (r=0.137, p=0.008; r=0.176, p=0.0006, respectively) (Table 5). The midterm exam results were excluded from the correlation study as it was carried out before implementing the TBL/OBE. Despite this weak correlation, there is a significantly higher achievement in TBL/OBE when compared to their achievement in the final and total exam results (Figure 5).

**Table 5:** Correlation of the students' performance in TBL/OBE and the overall performance in the clinical biochemistry course. TBL/OBE: team-based learning and open-book exam; r: correlation coefficient; p-value <0.05 is considered significant

Assessment	TBL/OBE vs final	TBL/OBE vs total
r	0.1368	0.1759
p-value	0.0081	0.0006

## Discussion

The current study used a combined model of modified TBL/OBE as learning and assessment strategies in clinical biochemistry for medical students. Assessment is an integral part of education, and teachers are concerned about using reliable and appropriate assessment methods [12]. They aim to ensure the integrity, clarity, reliability, and validity of their assessment methods [13,17].

When correlating students' performance in TBL/OBE with their academic success, significant positive associations were observed with both the final and overall examination scores in the clinical biochemistry course, corroborating the findings of Ahmed et al. [18]. Their research examined the impact of TBL on students' scores in basic medical science examinations. Their study's outcomes validated our theoretical understanding by determining that TBL is linked with elevated scores, enhancing the learning experience and knowledge retention. Our results align with those from that study, namely, that TBL could enhance critical thinking, self-confidence, and problem-solving skills. Unlike ours, their study lacks the combination of a group activity (the modified TBL implemented in our study) with the OBE.

This study implemented the traditional sit-down OBE [19] to test its usefulness at the educational level, either for learning or as an assessment method. It is less stressful to prepare for OBEs and resemble clinical practice more closely, as it is generally easy to obtain information during consultations. Furthermore, OBEs encourage deeper learning and assessment of higher-level outcomes. Moreover, it has been reported that the introduction of "open-resource" exams has positively impacted the health professions [20].

OBEs allow students to demonstrate their level of knowledge and understanding in the allocated time. Moreover, OBEs may contribute to developing students' higher-level skills, including conceptualization, problem-solving, and reasoning [5].

To the best of our knowledge, this is the first study that has integrated an OBE and the modified TBL as learning and assessment tools in the clinical biochemistry curriculum. We were therefore interested in thoroughly evaluating the students' perception of this experience.

The students reported a remarkable impact of the TBL/OBE on the learning materials and specific objectives of the subject. The students were allowed sufficient time (10 days) to prepare before the TBL/OBE session. In addition to uploading prerecorded lectures and videos, the material and learning resources related to the topic (metabolic integration and interrelation) were determined. Interestingly, 58% of students indicated they needed more time to prepare for the OBE than for CBE. This finding differs from previously reported data showing that OBEs reduce studying and preparation time among other university students in similar studies [21,22]. This finding could be explained by the fact that the students in our institute need more experience and training in this type of assessment. Indeed, with OBEs, students need more time to access textbooks, notes, and other course-related resources before the exam. In addition, students spend considerable time searching for answers instead of formulating their solutions.

In the current study, we implemented the team-based method, with cooperation between the students to answer the exam questions during the closed- and open-book sessions.

The students' perceptions of the group teamwork activity (modified TBL) varied. The majority of the students worked cooperatively, a minority worked competitively, and a negligible percentage worked individually. Moreover, they reported that they enjoyed the group discussion, teamwork, and OBE experience. We suggest this is based on their ambition to secure better grades, which collaborative efforts can help attain. Additionally, it's important to note that students with lower performance, particularly those receiving grades D and F, are more inclined toward teamwork. A study by Parthasarathy et al. has shown that students agree that they were determined to contribute to their team's learning and found the teamwork activity more engaging and less distracting [23]. Moreover, team learning encourages self-reading, holds students accountable for their learning, triggers team discussions [24], increases self-confidence among students, and creates a better learning environment [25].

The training of medical students to work in teams is an essential target of the implementation of the OBE in our study. Developing teamwork skills before graduation is critical for a well-functioning and effective healthcare system, as such skills are essential to provide the best management and full assistance to patients and their families [26].

The students who worked competitively and those who did not participate in the group activity could reflect a lack of experience in teamwork and TBL [23]. Moreover, this phenomenon could be explained by individual personality variability, as introverted students usually prefer to avoid teamwork [27,28].

The present study examined the difficulty index and discrimination factors of the questions in the open-book and the closed-book formats. On the one hand, the OBE had a significantly higher difficulty index than CBE. On the other hand, the discrimination power of the CBE was significantly greater than that of the open-book format, indicating that OBEs are easier, while CBEs are more discriminating.

OBEs are expected to be easier, given that they do not assess students' memorization capacity and enable students to find answers stress-free [29]. In addition, students perceive OBEs as an easy exam with reduced anxiety [7] since the accessible consulting of authorized resources enables the students to find the answers, ideally without any stress [30]. These advantages make the OBE easier.

In our model, all the answers were open-ended despite being primarily presented as multiple-choice questions. The students needed to explain how they selected the best correct answer, which was the goal. The students succeeded in applying knowledge, analysis, and interpretation in their choices in the form of a short response. This skill showed the students' reasoning skills and challenged their thoughts [31].

Neumann et al. [32] reported good discrimination power between high- and low-performing students in the OBE. Our results confirmed their findings and showed a higher discrimination ability for CBEs. However, unlike our study, Neumann et al. did not compare their findings regarding the OBE to the CBE.

With CBEs, students spend more time and exert greater mental effort to answer exams, which emphasizes that high-performing medical students are more closely linked to well-organized subject knowledge than low-performing students [7]. CBEs require rapid retrieval of stored information and rote memorization [33,34], which is a skill that is developed to a greater extent in high performers and, hence, improves CBE discrimination.

To confirm our hypothesis, we tested the perception of students with different grade levels of OBE compared to CBE. Surprisingly, students with A and B grades preferred the CBE format. In addition, students with B grades preferred to work competitively, and, compared to other grade groups, fewer students with A grades (50%) enjoyed the OBE experience and teamwork compared to 80% with F grades.

CBE encourages deep learning, which is predominantly compulsory for memorizing and retaining knowledge. Thus, students with A and B grades feel more confident when preparing for closed-book tests [35], which explains why high achievers (students with A and B grades) preferred CBEs over OBEs. Moreover, these students are more motivated to study for closed-book tests than OBEs.

Moreover, the high discrimination power of CBEs compared to OBEs may be why high-achieving students prefer it, to satisfy their competitive nature regarding academic achievements and scores. However, their competitive spirit and preferences did not interfere with their enjoyment of the OBE and their expression of this perception.

Finally, we examined the differences in the perceptions of males and females. Male students reported that the OBE questions are more manageable, are less stressful, and require less time to prepare for, even though the majority of the students, both males and females, worked cooperatively.

This finding could be partially explained by the fact that male students perform well when examined under challenge (the challenge in our case was the search for answers in the learning material and web during the OBE). In contrast, the females showed a decrease in performance as pressure increased [36]. This could explain the relationship between the gender gap and academic performance, which is attributable to pressure [37], as male students overtake female students at high-pressure levels. Moreover, it has been reported that male students are capable of more remarkable adjustments and adaptations in their perception of OBE questions and other types of exams in contrast to female students [38,39].

In a systematic review, Johanns et al. [40] discuss the debate regarding the use of OBEs instead of CBEs for assessments and present both advantages and disadvantages. They conclude that the OBE is a powerful teaching and assessment tool, as it has been proven to enhance critical thinking, the analysis of ideas, and the power to integrate concepts to aid in solving problems; nevertheless, it is not in common use in many health professional institutes. However, compared to our study, the study by Johanns et al. lacks integration with a group activity to answer the question, which can be considered unique to our research [40].

The practical implication of the collaborative TBL/OBE method in clinical biochemistry is that it benefits gaining information and understanding and expands knowledge content. Students understood the meaning of the questions through group discussions, as all group members engaged in the debate and efficiently shared their ideas through close communication. Moreover, they all provided and received immediate feedback and fully participated in solving complex tasks. Formulating the correct answer was a task for all, meaning they were keen to complement each other's efforts rather than compete. Moreover, the practical implementation of the OBE enhanced the students' learning and removed tension. We attempted to prepare them for the open-book world they would encounter during their career with operational decision-making. Increasing their confidence in their capability to work under challenging conditions and improving their grasp of the concept of the problem and how to overcome it through the open-book method in collaboration with their colleagues and workmates are essential theoretical and practical implications of the current study.

Other significant implications could be considered. The first implication is that a mixed assessment tool should be used for student evaluation. A hybrid method, such as the combined OBE/TBL in our study, could be beneficial as many skills can be evaluated simultaneously (communication, critical thinking, problem-solving, teamwork, decision-making, etc.). The second implication is the potential use of the assessment as a powerful learning tool rather than only for evaluation. Many of the hidden capabilities of students are disclosed and discovered at exam time. These capabilities help them gain knowledge and critical thinking abilities, making retaining knowledge easier.

The current study encountered some limitations, which include the low number of questions in both exams (OBE and CBE). The limited time of the exam affected its regulation, so we were forced to use the same questions in the OBE and CBE. Moreover, we used a modified format of TBL, in which iRAT was not implemented. Finally, there was only a single session of TBL/OBE, so its impact on students' grades and overall comprehension could not be thoroughly studied. While the study assessed immediate perceptions and outcomes, examining the long-term retention of knowledge gained through TBL/OBE versus traditional methods would be valuable. This could involve follow-up tests or assessments several months after completing the course.

## Conclusions

The current study concludes that a combined model of modified TBL/OBE is a successful learning and assessment strategy in clinical biochemistry for medical students based on the students' perceptions. The students' perception of an OBE with teamwork was markedly different from that of the CBE. Compared to the females, there was a greater preference for OBEs among male students. Moreover, the CBE has a high discrimination power, meaning high-achieving students favor the CBE.

Based on these considerations, we recommend extending the implementation of the combined model of TBL/OBE over the clinical biochemistry course, in addition to its use as a learning and assessment strategy in other medical students' subjects. Moreover, we recommend implementing different methods of cognitive-based learning and assessment strategies in medical and health professional education.

For faculty considering implementing TBL and OBE, it's crucial to undergo specialized training in these methodologies, ensuring alignment with course objectives. Embracing educational technologies, such as collaborative online tools, enhances the TBL/OBE experience.

## References

[1] Alamoudi A, Hassanien M, Al Shawwa L, Bima A, Gad H, Tekian A (2018). Introducing TBL in clinical biochemistry: perceptions of students and faculty. MedEdPublish.

[2] Alamoudi AA, Al Shawwa LA, Gad H, Tekian A (2021). Team-based learning versus traditional didactic lectures in teaching clinical biochemistry at King Abdulaziz University; learning outcomes and student satisfaction. Biochem Mol Biol Educ.

[3] Elsamanoudy AZ, Fayez FA, Alamoudi A, Awan Z, Bima AI, Ghoneim FM, Hassanien M (2021). Project-based learning strategy for teaching molecular biology: a study of students' perceptions. Educ Med J.

[4] Mahmoudzadeh-Sagheb H, Heidari Z, Mohammadi M (2015). A survey of students' perspectives of open-book examinations in histology/embryology. J Med Educ.

[5] Green SG, Ferrante CJ, Heppard KA (2016). Using open-book exams to enhance student learning, performance, and motivation. Journal of Effective Teaching in Higher Education.

[6] Larwin KH, Gorman J, Larwin DA (2013). Assessing the impact of testing aids on post-secondary student performance: a meta-analytic investigation. Educ Psychol Rev.

[7] Durning SJ, Dong T, Ratcliffe T, Schuwirth L, Artino AR Jr, Boulet JR, Eva K (2016). Comparing open-book and closed-book examinations: a systematic review. Acad Med.

[8] Sam AH, Reid MD, Amin A (2020). High-stakes, remote-access, open-book examinations. Med Educ.

[9] Davies DJ, McLean PF, Kemp PR (2022). Assessment of factual recall and higher-order cognitive domains in an open-book medical school examination. Adv Health Sci Educ Theory Pract.

[10] Sweller J, van Merrienboer JJ, Paas FG (1998). Cognitive architecture and instructional design. Educ Psychol Rev.

[11] Ghanbari S, Haghani F, Barekatain M, Jamali A (2020). A systematized review of cognitive load theory in health sciences education and a perspective from cognitive neuroscience. J Educ Health Promot.

[12] Awad Ahmed FR, Ahmed TE, Saeed RA, Alhumyani H, Abdel-Khalek S, Abu-Zinadah H (2021). Analysis and challenges of robust E-exams performance under COVID-19. Results Phys.

[13] Hargreaves E (2007). The validity of collaborative assessment for learning. Assess Educ.

[14] Zhang XC, Lee H, Rodriguez C, Rudner J, Papanagnou D (2018). A novel approach to debriefing medical simulations: the six thinking hats. Cureus.

[15] Shimizu I, Kikukawa M, Tada T, Kimura T, Duvivier R, van der Vleuten C (2020). Measuring social interdependence in collaborative learning: instrument development and validation. BMC Med Educ.

[16] Blumenfeld PC, Marx RW, Soloway E, Krajcik J (1996). Learning with peers: from small group cooperation to collaborative communities. Educ Res.

[17] Gamage KA, Silva EK, Gunawardhana N (2020). Online delivery and assessment during COVID-19: safeguarding academic integrity. Educ Sci.

[18] Ahmed M, Athar S, Zainab S, Akbani S, Hasan B, Hameed U (2022). Does team-based learning affect test scores of the basic medical sciences students in a modular curriculum?. Int J Health Sci (Qassim).

[19] Shakeel A, Shazli T, Salman MS, Naqvi HR, Ahmad N, Ali N (2021). Challenges of unrestricted assignment-based examinations (ABE) and restricted open-book examinations (OBE) during COVID-19 pandemic in India: an experimental comparison. Hum Behav Emerg Technol.

[20] Teodorczuk A, Fraser J, Rogers GD (2018). Open book exams: a potential solution to the "full curriculum"?. Med Teach.

[21] Theophilides C, Koutselini M (2010). Study behavior in the closed-book and the open-book examination: a comparative analysis. Educ Res Eval.

[22] Rakes GC (2008). Open book testing in online learning environments. J Interact Online Learn.

[23] Parthasarathy P, Apampa B, Manfrin A (2019). Perception of team-based learning using the team-based learning student assessment instrument: an exploratory analysis within pharmacy and biomedical students in the United Kingdom. J Educ Eval Health Prof.

[24] Parmelee D, Michaelsen LK, Cook S, Hudes PD (2012). Team-based learning: a practical guide: AMEE guide no. 65. Med Teach.

[25] Huilaja L, Bur E, Jokelainen J, Sinikumpu SP, Kulmala P (2022). The effectiveness and student perceptions of peer-conducted team-based learning compared to faculty-led teaching in undergraduate teaching. Adv Med Educ Pract.

[26] de Oliveira EM, Spiri WC (2006). Family Health Program: the experience of a multiprofessional team [Article in Portuguese]. Rev Saude Publica.

[27] Persky AM, Henry T, Campbell A (2015). An exploratory analysis of personality, attitudes, and study skills on the learning curve within a team-based learning environment. Am J Pharm Educ.

[28] Bradley CL, Jeter E, Lee S, Cooper JB (2021). A teamwork workshop to improve pharmacy students' growth mindset and communication skills. Am J Pharm Educ.

[29] Rehman J, Ali R, Afzal A, Shakil S, Sultan AS, Idrees R, Fatima SS (2022). Assessment during Covid-19: quality assurance of an online open book formative examination for undergraduate medical students. BMC Med Educ.

[30] Myyry L, Joutsenvirta T (2015). Open-book, open-web online examinations: developing examination practices to support university students' learning and self-efficacy. Active Learn High Educ.

[31] Fuller R, Joynes V, Cooper J, Boursicot K, Roberts T (2020). Could COVID-19 be our 'There is no alternative' (TINA) opportunity to enhance assessment?. Med Teach.

[32] Neumann J, Simmrodt S, Teichert H, Gergs U (2021). Comparison of online tests of very short answer versus single best answers for medical students in a pharmacology course over one year. Educ Res Int.

[33] Dave M, Dixon C, Patel N (2021). An educational evaluation of learner experiences in dentistry open-book examinations. Br Dent J.

[34] Hong S, Go B, Rho J, An S, Lim C, Seo DG, Ihm J (2023). Effects of a blended design of closed-book and open-book examinations on dental students' anxiety and performance. BMC Med Educ.

[35] Malone DT, Chuang S, Yuriev E, Short JL (2021). Effect of changing from closed-book to formulary-allowed examinations. Am J Pharm Educ.

[36] Montolio D, Taberner PA (2021). Gender differences under test pressure and their impact on academic performance: a quasi-experimental design. J Econ Behav Organ.

[37] Iriberri N, Rey-Biel P (2019). Competitive pressure widens the gender gap in performance: evidence from a two-stage competition in mathematics. Econ J.

[38] Arthur N, Everaert P (2012). Gender and performance in accounting examinations: exploring the impact of examination format. Account Educ.

[39] Fallan L, Opstad L (2014). Beyond gender performance in accounting: does personality distinction matter?. Account Educ.

[40] Johanns B, Dinkens A, Moore J (2017). A systematic review comparing open-book and closed-book examinations: evaluating effects on development of critical thinking skills. Nurse Educ Pract.

